# Interventions to help coral reefs under global change—A complex decision challenge

**DOI:** 10.1371/journal.pone.0236399

**Published:** 2020-08-26

**Authors:** Kenneth R. N. Anthony, Kate J. Helmstedt, Line K. Bay, Pedro Fidelman, Karen E. Hussey, Petra Lundgren, David Mead, Ian M. McLeod, Peter J. Mumby, Maxine Newlands, Britta Schaffelke, Kerrie A. Wilson, Paul E. Hardisty

**Affiliations:** 1 Australian Institute of Marine Science, QLD, Australia; 2 School of Biological Sciences, The University of Queensland, St. Lucia, QLD, Australia; 3 ARC Centre of Excellence in Mathematical and Statistical Frontiers, School of Mathematical Sciences, Queensland University of Technology, QLD, Australia; 4 Centre for Policy Futures, The University of Queensland, QLD, Australia; 5 Great Barrier Reef Foundation, QLD, Australia; 6 TropWATER, James Cook University, QLD, Australia; 7 ARC Centre of Excellence for Environmental Decisions, The University of Queensland, QLD, Australia; Academia Sinica, TAIWAN

## Abstract

Climate change is impacting coral reefs now. Recent pan-tropical bleaching events driven by unprecedented global heat waves have shifted the playing field for coral reef management and policy. While best-practice conventional management remains essential, it may no longer be enough to sustain coral reefs under continued climate change. Nor will climate change mitigation be sufficient on its own. Committed warming and projected reef decline means solutions must involve a portfolio of mitigation, best-practice conventional management and coordinated restoration and adaptation measures involving new and perhaps radical interventions, including local and regional cooling and shading, assisted coral evolution, assisted gene flow, and measures to support and enhance coral recruitment. We propose that proactive research and development to expand the reef management toolbox fast but safely, combined with expedient trialling of promising interventions is now urgently needed, whatever emissions trajectory the world follows. We discuss the challenges and opportunities of embracing new interventions in a race against time, including their risks and uncertainties. Ultimately, solutions to the climate challenge for coral reefs will require consideration of what society wants, what can be achieved technically and economically, and what opportunities we have for action in a rapidly closing window. Finding solutions that work for coral reefs and people will require exceptional levels of coordination of science, management and policy, and open engagement with society. It will also require compromise, because reefs will change under climate change despite our best interventions. We argue that being clear about society’s priorities, and understanding both the opportunities and risks that come with an expanded toolset, can help us make the most of a challenging situation. We offer a conceptual model to help reef managers frame decision problems and objectives, and to guide effective strategy choices in the face of complexity and uncertainty.

## Introduction

Climate change is impacting tropical coral reefs globally. Solutions are needed urgently to help reefs cope—and for three reasons. First, coral reefs are biologically the richest ecosystem in the world’s oceans [1,2]. Second, they provide ecosystem services that support livelihoods, recreation and economic activities worth hundreds of billions of dollars annually [3–6]. Third, coral reefs are among the most climate-sensitive ecosystems on Earth [7,8].

The recent marine heat wave exacerbated by the 2015/16 El Niño event led to extensive episodes of coral bleaching [9,10]. On Australia’s Great Barrier Reef, back-to-back bleaching in 2016 and 2017 led to unprecedented loss of coral cover [11,12]. While corals, the reef ecosystem engineers, can recover from severe disturbances [13], the projected shortening of interludes between increasingly severe bleaching events under even optimistic climate futures [14,15] will diminish the scope for net reef recovery. Growing pressure from ocean acidification, a chemical consequence of carbon emissions, will further diminish this scope [16].

Reducing greenhouse gas emissions will be necessary to sustain coral reefs in the long term. However, global emissions increased in 2017, 2018 and 2019 [17,18]. Current unconditional climate-mitigation pledges would see the world warm by 2.9 to 3.4°C above pre-industrial levels this century [19]. Even if global warming could be kept below 1.5°C–currently with less than 1% chance given pledges [20]–the surface waters of tropical oceans would warm another 0.3°C in coming decades [16]. Even such minimal continued warming would damage the sensitive coral species [21] that drive reef recovery [22] and form critical habitats [23]. Thus, as it currently stands, the Paris Accord will not protect coral reefs.

Another avenue is to build ecosystem resilience by further improving conventional management interventions and their governance [6]. Reducing nutrient pollution [24,25], limiting herbivore overfishing [26] and removing coral predators [27] can support resilience by enhancing coral growth and survival. This is so because (i) sediments have direct negative effects on coral recruitment and growth [28,29], and (ii) nutrient run-off in combination with herbivore overfishing reduce coral resilience by favouring the growth and survival of algae which prevent coral recruitment [30,31]. Reducing nutrient run-off may also reduce bleaching risks [32–34] and dampen outbreak risks of coral-eating crown-of-thorns starfish [35]. A problem, however, is that climate change—in addition to causing increased mortality via bleaching events [11] and storms [36]—erodes two key biological processes that underpin coral resilience: growth rate [37,38] and recruitment rate [39,40]. Thus, increasing conventional management action cannot compensate for the climate-driven decline in coral survival, growth and recruitment of many coral species in many places [16,22,41,42]. The situation is analogous to that of a cancer patient: good care helps, but it is only a solution when combined with a cure.

Both climate mitigation and intensified conventional management are indispensible to sustaining healthy coral reefs into the future. But more is needed. While natural processes of physiological acclimation may improve coral heat tolerance [43,44] genetic adaptation generally acts on longer timescales [45]. Warm-adapted traits may not spread fast enough in most coral species to keep up with the rate of global warming, even under strong carbon mitigation [14,46–48].

To build the biological resilience required to tolerate and recover from the projected escalation of marine heat waves [49] and increasing pressure from ocean acidification [50], high rates of coral adaptation will be needed. Active interventions to assist adaptation include ways to enhance coral performance including thermal tolerance [51–53] and/or lowering the exposure of corals to bleaching conditions–i.e. dampening heat waves locally and shading against strong solar radiation. A recent review by the National Academy of Sciences, Engineering and Medicine identified 23 candidate interventions with varying scope to become effective, feasible and safe [54]. While such measures are often referred to as restoration, they go beyond classical restoration techniques by altering biological and ecological resilience or stress exposure, or both. A similar review completed for the Australian Government’s Reef Restoration and Adaptation program (RRAP) examined 160 such interventions across a range of scales (from a few square metres to hundreds of reefs), concluding that 43 warranted more research and development (Box 1) and that the possibilities for positive impact overall were promising enough to warrant further investment [55].

## Box 1. Categories of intervention based on functional objective as used in the Reef Restoration and Adaptation Program (RRAP) on the Great Barrier Reef [55]

**Table:** 

Type	Function	Interventions include	Scale
1	Cooling and shading to reduce coral stress during heat waves	Cooling by mixing or pumping, and shading by cloud brightening, fogging, misting, microbubbles, thin surface films, algae or structures	Local (meters) to regional (1000s of kilometers)
2	Adding structures to provide habitat and to stabilise substrate to enhance recruitment	Rubble stabilisation by mesh, chemical or natural bonding, and the introduction of various types of structures or frames	Local only (meters to hectares)
3	Enhance recruitment of warm-adapted corals to enhance resilience	Translocation of larval slicks and relocation of corals in situ (assisted gene flow), coral propagation of all life-history stages using aquaculture methods	Local (meters or hectares) to sub-regional (tens of kilometers)
4	Bio-control to support coral reef resilience	Control of algae and other species which inhibit coral growth and reproduction	Local only (meters to hectares)
5	Coral treatments	Support coral health and survival using probiotics, feeding, medicine or other treatments	Local only (meters to hectares)
6	Supporting natural adaptation	Increase thermal tolerance of natural coral populations via selective breeding	Local but with capacity for regional impact via connectivity
7	Enhancing adaptation using new technologies	Increase thermal tolerance of corals using synthetic biology and gene-engineering/editing approaches	Local but with capacity for regional impact via connectivity

**Fig 1:**
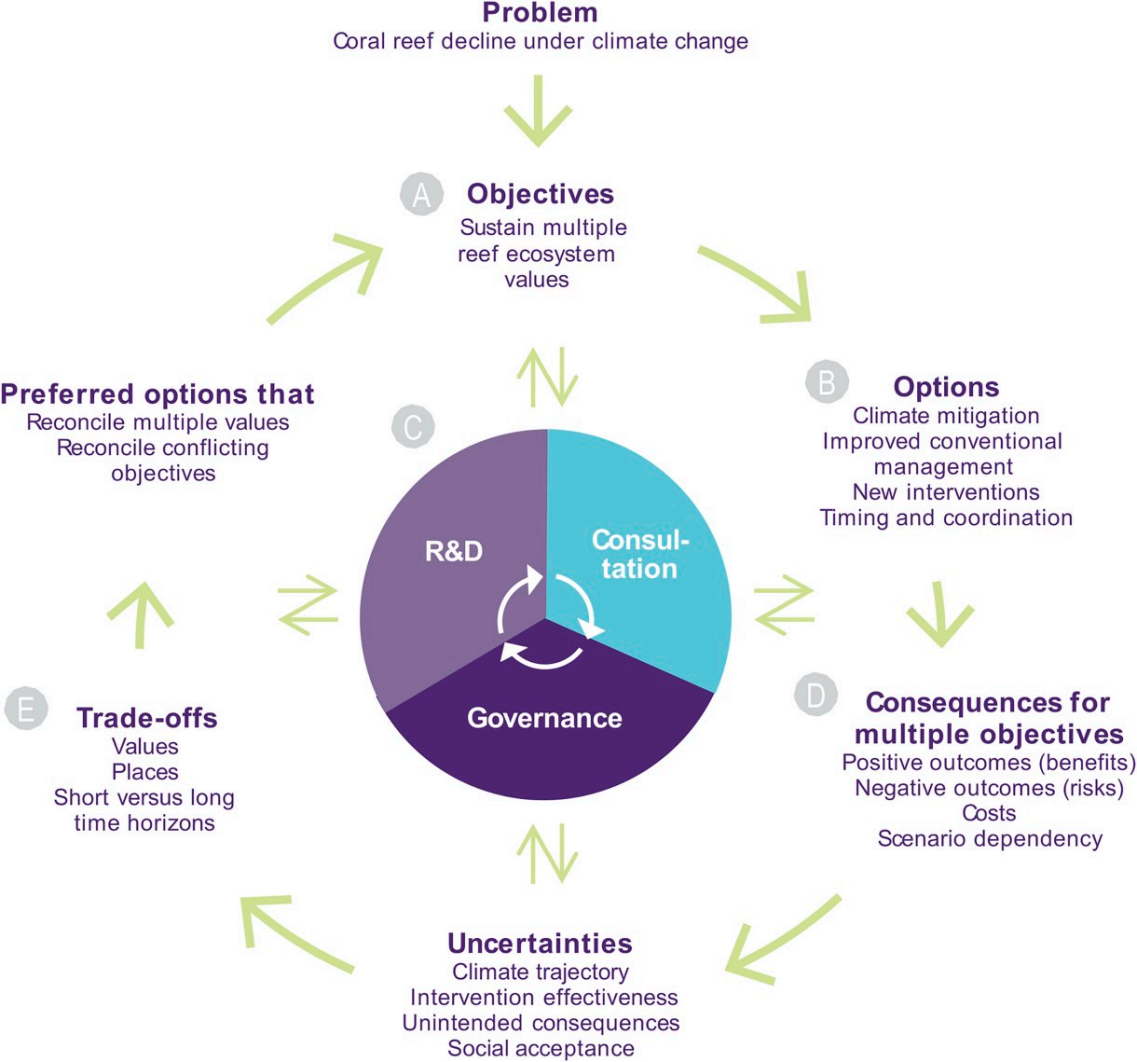
Structured decision-making framework applied to the coral reef crisis under climate change. The framework is centred on an adaptive management cycle of intervention research and development (R&D), stakeholder and regulatory consultation, and governance. Two-way arrows indicate that steps in the structured decision-making framework form adaptive links with R&D, consultation and the governance of how resources are allocated and actions implemented given updated information. Adapted from: [56–58].

The questions are then: what new interventions should be developed and added to the management toolbox for coral reefs? And once developed, when and where should they be deployed? How should performance expectations, risks and uncertainties be managed? We argue that an expanded intervention toolbox, as an adaptation strategy, presents at least three core challenges for reef managers, policy-makers and regulators: (1) framing the problem and setting the right objectives, (2) managing risks and uncertainties given the urgency, and (3) assessing and making necessary trade-offs (Fig 1). Here we address each of these challenges. We close with a discussion of how fast and effective research and development (R&D) strategies provide options in a time of crisis and how the governance of on-reef intervention will face unprecendented challenges of coordination and integration. We conclude that the sooner we step up to this challenge, the closer we will be to producing solutions.

### Challenge 1: Setting the right objectives to solve the right problem

Pristine coral reefs are no more [59,60]. Even under best-case emissions trajectories, coral reefs will likely be transformed by climate change [11], so striving to retain or recreate historical levels of biodiversity and richness in a warming world may be futile. The most a conservation program may hope to deliver are sustained, yet altered, ecosystem services and priority values. And the results of any program will ultimately depend on how successful emission reductions become. These considerations affect our problem framing and the objectives we can achieve (Fig 1A). For example, is the objective to stem the decline in reef biodiversity, is it to sustain ecosystem services, or perhaps to create new ones? Is the objective to stem the decline of key (prized) species, or to sustain the key ecological functions they underpin? Perhaps provocatively, is it really coral reefs we seek to sustain, or is it the benefits they provide for society? We can’t have one without the other, but asking the question helps clarify objectives, and ultimately what we are willing to trade off. Different answers to these questions would lead to very different reef conservation programs.

Defining multiple, and often conflicting, objectives for complex social-ecological systems such as coral reefs is challenging, but critical. Within objectives, which values can be sustained with the capabilities and resources available? Coral reefs produce numerous value streams to society [61,62]. Bona fide adaptation solutions would be those that strike a balance across such value streams–monetary and otherwise. Altered, but functionally resilient, ecosystems are increasingly being embraced in terrestrial and freshwater conservation programs [63–66]. The time may now be right to explore such options for coral reefs also. We revisit this challenge under *Prioritisation and trade-offs*. With a clear understanding of objectives and values, the decision-making process around developing and applying new and potentially contentious intervention options, in combination with mitigation and conventional management (Fig 1, step B), can become informed and transparent [57].

### Challenge 2: Balancing benefits and risks in the face of uncertainty

Developing new technologies for environmental management and conservation is risky: it is expensive, takes a long time, and success is not guaranteed. Risks associated with emerging technologies, whether perceived or real, and their potential side effects, costs, and uncertainties trigger precaution [67]. There is good reason for this as history is replete with examples of how interventionist management can result in destructive outcomes [68]. The managers who introduced cane toads to Australia in 1935 to manage the cane beetle did neither have experience nor foresight to consider the catastrophic invasive potential of the toads. Today, the scientific and regulatory communities are much more informed about the biological, ecological, ethical, legal and social implications of new and emerging technologies [69]. Examples of advancement in the management of risk and uncertainty across a diversity of fields include the protection of nature reserves against invasive species [70], managed readiness levels of new technologies that enter aviation and space programs [71], risk assessments of new drugs prior to approval [72,73] and the adoption of driverless cars [74]. Applied coral reef research and development can and should learn from these and other fields. Doing so can help identify options that, when implemented in a coordinated approach after rigorous development and consultation (Fig 1, step C), are effective, safe, acceptable to the public and regulators, and economically rational.

Critically, in a time of rapid climate change, being risk averse can be risky [75]. Delaying new interventions because of uncertainty around side effects could mean losing key species and functions. However, the risk associated with status quo under different climate futures must be balanced against the risk of premature intervention, especially with technologies that are not yet ready for deployment [76,77]. Premature deployment of untested interventions (e.g. genetic engineering, assisted migration, solar radiation management) may cause ecosystem disruptions [54,78,79]. The sooner research and development programs evaluate the potential risks and benefits of interventions, the more informed policy decisions can be about whether to deploy, delay, or dismiss an intervention. This approach is the basis for NASA’s assessment of readiness levels of new technologies entering space programs [80], for expanding the number of options for medical treatments [81], and most recently for Australia’s Reef Restoration and Adaptation Program for the Great Barrier Reef [55]. Unfortunately, the motivation and social license to start conservation programs typically come when ecosystems or species are already in advanced decline [75]. Such delayed action represents a lost opportunity as interventions take time to develop, and because damage-prevention and restoration are now both needed to sustain ecosystems [82,83]. For example, coral populations in the northern Great Barrier Reef (GBR) are adapted to 1–2°C higher temperatures than populations in the central section [84], but the North-to- South larval spread is limited by diverging currents [47,85]. Under expectations of escalated GBR-wide warming [86], building resilience in the central and south using warm-adapted coral stock from the north will be a race against time as both donor reefs and receiving reefs are at risk. While classical reef-restoration approaches using local coral stock or larvae may enhance reef recovery following disturbances [87,88], enhanced climate tolerance is needed to support coral resilience under climate change [54].

Precaution is central to policy and regulation, but social science research indicates the need to interpret and understand risks more broadly [68]. Risk assessments of new interventions need to consider views that go beyond those of scientists and regulatory experts. Thus, decision makers and management agencies need to consult reef stakeholders (e.g. tourism operators, commercial and recreational fisheries, conservation groups), Traditional Owners and the wider community. Risk assessments in this context need to be tackled at three levels (1) the risk regime of future climatic conditions, (2) whether interventions will really produce the intended benefits, and (3) risks and costs versus benefits of early vs delayed implementation (Fig 1, step D). Such assessments are complicated by the fact that different future conditions will require different solutions, timing and risk tolerance [53]. What would constitute premature intervention deployment under the expectation of 1.5°C warming this century could be too-little-too-late under the expectation of 3°C warming. Further, picking intervention solutions that are robust to climate change could be a blunt strategy because both timing and intervention type could be misaligned with the conditions that eventually unfold. The most effective solution from a risk-management perspective could be a combination of intervention hedging and improved forecasting, not unlike an investment portfolio strategy [89].

### Challenge 3: Prioritisation and tradeoffs–we can’t save everything

The gap between resources available and resources needed for conservation is widening [90,91]. Consequently, investment prioritisation is necessary [92,93]. How this is done needs to be anchored in the problem framing and by clearly defined ecological, economic and social objectives. Further, prioritisation needs to have line of sight to outcomes that can be achieved given climate uncertainty and funding contraints (Fig 1, step E). As an example, consider two extreme yet realistic prioritisation alternatives for a large reef system such as the Great Barrier Reef. Should we aim to sustain a minimum of 5% coral cover over a 1000 km^2^ area of reef, or a minimum of 25% coral cover over a 200 km^2^ area? Logistics will differ, but the net result is the same in terms of coral area sustained: 50 km^2^. However, depending on the spatial configuration of the saved corals, these alternatives would produce very different ecological outcomes and values for society. Spreading efforts across a large area would speak to system integrity and perhaps the Outstanding Universal Value of the Great Barrier Reef World Heritage Area [94]. Downsides of spreading efforts thinly include reduced capacity to sustain critical ecological functions such as net reef accretion [95], and reduced fitness via a reduced demographic Allee effect [96,97]. Conversely, concentrating efforts on a selection of just a few but glorious reefs could sustain parts or all of the GBR’s tourism industry, which is spatially concentrated [98]. It would enable managers to support ecological functions and services on those focal reefs more easily, and perhaps create spill-over effects to other reefs [99]. Taken to the extreme under severe climate change, spatial prioritisation under resource constraints could reduce the Great Barrier Reef to a fragmented (and therefore vulnerable) network of coral oases in an otherwise desolate seascape.

Other options might involve targeting reefs that are gateway nodes in the spatial reef network–in other words, investing in well-connected reefs located in the least thermally stressed environments [100]. Here, efforts to support population growth of climate-hardy corals on source reefs (larval donors) may allow export of their beneficial traits to reefs downcurrent through paths of natural dispersal [99,101]. But risks are that disease agents and potentially invasive species arising from either translocation or assisted gene flow may also spread via similar routes [47]. Selection criteria should thus favour the dispersal of desirable species only [99]. The decision challenge associated with spatial prioritisation is therefore one of maximising the spread of genes or traits that produce benefits and minimising those that represent risks. Another option may be to assemble a portfolio of reefs that have less risk of being exposed to the most damaging climate stressors [48,102]. Combining these options may both enhance resilience and reduce stress on priority reefs.

Prioritisation of species adds to the decision challenge for reef restoration and adaptation. Without significant climate mitigation, sensitive coral species will give way to naturally hardier ones [11], or to species that can adapt faster [45,103]. Picking who should be winners, and ultimately who will be losers, under continued but uncertain climate change is perhaps the biggest challenge facing R&D programs tasked with developing reef rescue interventions. Unfortunately, sensitive coral species tend to be the ones underpinning high-value ecosystem services, including habitat provision for a rich biodiversity [23] that in part underpin tourism [5]. Should we invest in making sensitive species hardier but risk failing by not making them hardy enough, thereby wasting resources? Or should we pursue a potentially less risky pathway and support the more climate-hardy species and help *them* adapt to the consequently altered ecosystems and the different goods and services they provide? Importantly, our best efforts to build coral resilience under severe climate change will not prevent reefs from transitioning to altered ecosystems [6,60]. Strategies to help humans adapt to a changed ecosystem need to combine with strategies that help reefs [104]. Lastly, can robust keystone species be found that can give climate protection to many other dependent species [105], thereby sustaining ecosystem services? The latter may ultimately be the most effective choice if species compositions allow the ecosystem to remain functionally resilient [64]. How these priorities are set ultimately depends on what society wants (objectives and values), what options can be achieved technically, institutionally and socially, and what compromises and risks we are willing to accept. The preferred strategy would be the one that delivers the most positive outcomes to priority objectives (and the values they encompass) with low or manageable risks and within resource constraints [57,58,106].

### R&D provides options, but choose carefully

The likelihood that the world will warm more than 2°C (air) since preindustrial levels this century was recently 95 percent [17,20]. With this outlook, new intervention options for coral reefs will be in growing demand. Importantly, however, no new intervention can be added to the operational management toolset without significant R&D; it is the prerequisite for intervention effectiveness, safety and cost efficiency [67]. How interventions are chosen for R&D and progressed through to deployment is both complex and critical because it will determine what options will ultimately be available for managers and when (Fig 1, steps B-E). Three questions are at the centre of reef intervention R&D. First, which interventions should be prioritised for development? Second, how should they be queued in time? Third, should intervention strategies be robust or targeted?

Limited resources for R&D means not all interventions can be assessed nor progressed. Complicating this problem is that the more the world warms, and the more ecosystems become affected, the greater the overall demand for intervention resources will be. Misguided investment choices can lock up vital resources in inferior solutions, hampering or preventing the development of superior ones [107]. Prioritising no-regrets options because they are inexpensive or less challenging technologically [77] could lead to regrets downstream by preventing or delaying the development of more effective solutions. Prioritisation of interventions for R&D should ideally be a fast adaptive process (indicated by multiple adaptive cycles in Fig 1) whereby combinations of interventions are continuously assessed for their combined benefits and risks against environmental, social and economic objectives [53]. In general terms, the right time to implement an intervention strategy following R&D would be when the cumulative (time-integrated) benefit-to-risk ratio of deployment exceeds the cumulative benefit-to-risk ratio risk of not deploying. Here, benefits are defined as positive outcomes (as likelihood and consequence) for ecosystem services and values for society, and risks as negative outcomes. The benefit-to-risk ratio of these contrasting strategies, however, will depend on the climate future (Fig 1, step D).

Robust strategies work across a range of climate change scenarios. While investing in a robust R&D strategy will give some return regardless of climate future, the strategy may eventually underperform because it trades off effectiveness for reduced risk. In contrast, targeted strategies are tuned to different climate scenarios. This involves betting on, and planning for, a specific climate trajectory. This represents high risk, but potentially also high reward. For example, a strategy that buys 1°C thermal tolerance for sensitive and valued coral species (on top of today’s 1°C global warming) may give high ecological, social and economic returns if global warming is kept below 2°C relative to preindustrial. If the world warms much more than 2°C, however, the strategy will be ineffective unless these species continue to adapt. Conversely, a strategy that bets on severe climate change and focuses on helping the hardiest species only (or develops artificial reefs) will miss the opportunity to protect biodiversity if a milder climate scemario unfolds in reality. Developing a portfolio of interventions that allows hedging and a staged roll-out of interventions as climate change unfolds may be ideal, but may again be constrained by resource availability for R&D, and the demands of urgency—real or perceived.

### Get people on board

Environmental problems are social problems [108]. Climate change, mass coral bleaching events and consequent coral reef decline are human-induced and require solutions from science and society. The dynamics of the current coral disease outbreak in the Caribbean are also consistent with ocean warming patterns [109–111]. While interventions that can build resistance to coral disease will differ from those that can build resistance to coral bleaching, a similar approach to solutions is needed. Solutions require innovative thinking and coordination between science, management and policy, and public engagement. There are concerns that restoration and adaptation are distractions from tackling global climate change, the main driver of coral reef decline [112]. Communication and engagement strategies must reinforce the message that restoration and adaptation are a health-care strategy that can only work in tandem with a cure: urgent global action to address climate change.

Any new interventions on coral reefs, in particular radical ones, will be up against hurdles to achieve social acceptance and to overcome regulatory constraints [104,105], leading to uncertainties that become barriers for solutions (Fig 1, step D). Existing regulations operate under a retrospective model that crowbars coral restoration and adaptation into existing policy and legislation. However, future policy development should accommodate risks of future climatic conditions (see challenge two above) whilst simultaneously adapting to the emerging opportunities and challenges of coral restoration and adaptation.

A handful of countries are currently developing or revising existing policy and regulatory processes to assist coral restoration and adaptation in the face of climate change: Australia, USA, Netherlands, France, Costa Rica, Japan, Columbia, and Thailand. The United Nations Environment Program have declared 2021–2030 as the UN Decade of Ecosystem Restoration. The aim is to “support and scale up efforts to prevent, halt and reverse the degradation of ecosystems worldwide and raise awareness of the importance of successful ecosystem restoration” [87].

To get people on board will require coordinated consulation and transparent decision making that considers all risks, benefits and value consequences of reef intervention in a structured way [106]. Open communication and engagement around objectives, options and trade-offs will be key.

### Strong and coordinated governance needed

Applying a coordinated and well-conceived coral reef intervention program which sets the right objectives, identifies and balances risks, and aims to make optimal tradeoffs, in the face of uncertainty while getting community buy-in and support, will depend on robust and appropriate governance [113, 114]. First, at the R&D stage, researchers will need to be provided with the resources to do the job, and the mandate to take risks. For something as new and potentially controversial as large-scale coral reef intervention, consultation and co-development mechanisms must involve regulators, reef stakeholders, Traditional Owners and the public. Internal processes must be agreed on at the outset, to allow ongoing, effective prioritisation while maintaining flexibility in the face of changing conditions and unexpected setbacks. Some of the tested interventions to be examined will simply not work. Strong governance will be particularly important if political pressure for quick action (just do something) mounts in the face of worsening climate conditions. Next, as R&D results yield prospective options for at-scale intervention, governance must adapt to a situation where the costs, profile, and risk associated with failure of the effort have grown substantially. Again, at this stage, costs, benefits, risks and community desires will have to be balanced and trade-offs made, for at-scale deployment to occur.

## Conclusions

An expanded toolbox of interventions will provide opportunities to build reef resilience against continued climate change. Without carbon mitigation, no intervention strategy will be successful in the long run. And no single intervention can produce adaptation solutions. A portfolio of new and existing interventions must be combined with mitigation.

New interventions come with risks, but so does the status quo. If the potential of new interventions can be unlocked and their benefits exceed risks for reef, people and economies, they should be developed and deployed. The challenge for science, management and policy, in consultation with communities, is to develop and adopt technologies that will be both safe and effective—and within years rather than decades.

What climate trajectory will unfold is uncertain. But what is certain is that we need an expanded set of options to safeguard coral reefs and dependent people and industries. Research and development can help, but only if efforts are focused, coordinated and highly integrated. To do this will require a level of organisation, collaboration and integration across disciplines never seen before in natural sciences, conservation and policy.

## References

[1] Knowlton N, Brainard RE, Fisher R, Moews M, Plaisance L, Caley MJ. Coral Reef Biodiversity. In: McIntyre AD, editor. Life in the World’s Oceans. Blackwell Publishing Ltd; 2010.

[2] Fisher R, Leary RAO, Low-Choy S, Mengersen K, Knowlton N, Brainard RE, et al Species richness on coral reefs and the pursuit of convergent global estimates. Curr Biol. 2015;25: 500–505. 10.1016/j.cub.2014.12.022 25639239

[3] Costanza R, de Groot R, Sutton P, van der Ploeg S, Anderson SJ, Kubiszewski I, et al Changes in the global value of ecosystem services. Glob Environ Chang. 2014;26: 152–158. 10.1016/j.gloenvcha.2014.04.002

[4] Hoegh-Guldberg O. Reviving the Ocean Economy: the case for action. Gland, Geneva, Switzerland; 2015.

[5] Spalding M, Burke L, Wood SA, Ashpole J, Hutchison J, zu Ermgassen P. Mapping the global value and distribution of coral reef tourism. Mar Policy. 2017;82: 104–113. 10.1016/j.marpol.2017.05.014

[6] Rogers A, Harborne AR, Brown CJ, Bozec Y, Castro C, Chollett I, et al Anticipative management for coral reef ecosystem services in the 21st century. Glob Chang Biol. 2015;21: 504–514. 10.1111/gcb.12725 25179273

[7] Walther G, Post E, Convey P, Menzel A, Parmesan C, Beebee TJC, et al Ecological responses to recent climate change. Nature. 2002;416: 389–395. 10.1038/416389a 11919621

[8] Hoegh-Guldberg O, Poloczanska ES, Skirving W, Dove S. Coral reef ecosystems under climate change and ocean acidification. Front Mar Sci. 2017;4: 1–20. 10.3389/fmars.2017.00158

[9] Heron SF, Eakin CM, Douvere F. Impacts of climate change on World Heritage coral reefs: a first global scientific assessment. Paris: UNESCO World Heritage Centre; 2017.

[10] Hughes TP, Anderson KD, Connolly SR, Heron SF, Kerry JT, Lough JM, et al Spatial and temporal patterns of mass bleaching of corals in the Anthropocene. Science. 2018;5: 80–83. 10.1126/science.aan8048 29302011

[11] Hughes TP, Kerry JT, Baird AH, Connolly SR, Dietzel A, Eakin CM, et al Global warming transforms coral reef assemblages. Nature. 2018;556: 492 10.1038/s41586-018-0041-2 29670282

[12] AIMS. Long-term Reef Monitoring Program—Annual Summary Report on coral reef condition for 2017/18. Townsville; 2018. Available: https://www.aims.gov.au/reef-monitoring/gbr-condition-summary-2017-2018

[13] Connell JH, Hughes TP, Wallace CC. a 30-Year Study of Coral Abundance, Recruitment, and Disturbance At Several Scales in Space and Time. Ecol Monogr. 1997;67: 461–488. 10.1890/0012-9615(1997)067[0461:AYSOCA]2.0.CO;2

[14] Frieler K, Meinshausen M, Golly a., Mengel M, Lebek K, Donner SD, et al Limiting global warming to 2 °C is unlikely to save most coral reefs. Nat Clim Chang. 2012;2: 1–6. 10.1038/nclimate1674

[15] King AD, Karoly DJ, Henley BJ. Australian climate extremes at 1.5°C and 2°C of global warming. Nat Clim Chang. 2017;7: 412–416. 10.1038/nclimate3296

[16] Anthony KRN. Coral reefs under climate change and ocean acidification: challenges and opportunities for management and policy. Annu Rev Environ Resour. 2016;41: 59–81. 10.1146/annurev-environ-110615-085610

[17] Jackson RB, Le Quéré C, Andrew RM, Canadell JG, Korsbakken JI, Liu Z, et al Global energy growth is outpacing decarbonization. Environ Res Lett. 2018;13: 120401 10.1088/1748-9326/aaf303

[18] Friedlingstein P, Jones MW, O’Sullivan M, Andrew RM, Hauck J, Peters GP, et al Global Carbon Budget 2019. Earth Syst Sci Data. 2019;11: 1783–1838. 10.5194/essd-11-1783–2019

[19] WMO. United In Science: High-level synthesis report of latest climate science information convened by the Science Advisory Group of the UN Climate Action Summit 2019. 2020.

[20] Raftery AE, Zimmer A, Frierson DMW, Startz R, Liu P. Less than 2°C warming by 2100 unlikely. Nat Clim Chang. 2017;7: 637–641. 10.1038/nclimate3352 30079118 PMC6070153

[21] Ainsworth TD, Heron SF, Ortiz JC, Mumby PJ, Grech A, Ogawa D, et al Climate change disables coral bleaching protection on the Great Barrier Reef. Science. 2016;352: 338–342. 10.1126/science.aac7125 27081069

[22] Wolff NH, Mumby PJ, Devlin M, Anthony KRN. Vulnerability of the Great Barrier Reef to climate change and local pressures. Glob Chang Biol. 2018;24: 1978–1991. 10.1111/gcb.14043 29420869

[23] Jones GP, McCormick MI, Srinivasan M, Eagle J V. Coral decline threatens fish biodiversity in marine reserves. Proc Natl Acad Sci. 2004;101: 8251–8253. 10.1073/pnas.0401277101 15150414 PMC419589

[24] Fabricius KE. Factors determining the resilience of coral reefs to eutrophication: a review and conceptual model. In: Dubinsky Z, Stambler N, editors. Coral reefs: an ecosystem in transition. Springer Netherlands; 2011 pp. 493–505. 10.1007/978-94-007-0114-4_28

[25] Ortiz J-C, Wolff NH, Anthony KRN, Devlin M, Lewis S, Mumby PJ. Impaired recovery of the Great Barrier Reef under cumulative stress. Sci Adv. 2018;4: eaar6127 10.1126/sciadv.aar6127 30035217 PMC6051737

[26] Steneck RS, Arnold SN, Boenish R, de León R, Mumby PJ, Rasher DB, et al Managing recovery resilience in coral reefs against climate-induced bleaching and hurricanes: a 15-year case study from Bonaire, Dutch Caribbean. Front Mar Sci. 2019;6: 1–12. 10.3389/fmars.2019.00265

[27] Shaver EC, Burkepile DE, Silliman BR. Local management actions can increase coral resilience to thermally-induced bleaching. Nat Ecol Evol. 2018;2: 1075–1079. 10.1038/s41559-018-0589-0 29915342

[28] Humanes A, Ricardo GF, Willis BL, Fabricius KE, Negri AP. Cumulative effects of suspended sediments, organic nutrients and temperature stress on early life history stages of the coral Acropora tenuis. Sci Rep. 2017;7: 1–11. 10.1038/s41598-016-0028-x 28281658 PMC5345069

[29] Wakwella A, Mumby P, Roff G. Sedimentation and overfishing drive changes in early succession and coral recruitment. Proc R Soc B. 2020; Forthcoming. 10.1098/rspb.2020.2575 PMC7779507 33323081

[30] Mumby PJ, Hastings A, Edwards HJ. Thresholds and the resilience of Caribbean coral reefs. Nature. 2007;450: 98–101. 10.1038/nature06252 17972885

[31] Steneck RS, Arnold SN, Mumby PJ. Experiment mimics fishing on parrotfish: Insights on coral reef recovery and alternative attractors. Mar Ecol Prog Ser. 2014;506: 115–127. 10.3354/meps10764

[32] Wooldridge SA, Done TJ. Improved water quality can ameliorate effects of climate change on corals. Ecol Appl. 2009;19: 1492–1499. 10.1890/08-0963.1 19769097

[33] Cunning R, Baker AC. Excess algal symbionts increase the susceptibility of reef corals to bleaching. Nat Clim Chang. 2013;3: 259–262. 10.1038/nclimate1711

[34] Donovan MK, Adam TC, Shantz AA, Speare KE, Munsterman KS, Rice MM, et al Nitrogen pollution interacts with heat stress to increase coral bleaching across the seascape. Proc Natl Acad Sci. 2020; 201915395 10.1073/pnas.1915395117 32094188 PMC7071909

[35] Fabricius KE, Okaji K, De’ath G. Three lines of evidence to link outbreaks of the crown-of-thorns seastar Acanthaster planci to the release of larval food limitation. Coral Reefs. 2010;29: 593–605. 10.1007/s00338-010-0628-z

[36] Cheal AJ, MacNeil MA, Emslie MJ, Sweatman H. The threat to coral reefs from more intense cyclones under climate change. Glob Chang Biol. 2017;23: 1511–1524. 10.1111/gcb.13593 28139035

[37] Anthony KRN, Kline DI, Diaz-Pulido G, Dove S, Hoegh-Guldberg O. Ocean acidification causes bleaching and productivity loss in coral reef builders. Proc Natl Acad Sci. 2008;105: 17442–17446. 10.1073/pnas.0804478105 18988740 PMC2580748

[38] Reynaud S, Leclercq N, Romaine-Liuod S, Ferrier-Pages C, Jaubert J, Gattuso J-P. Interacting effects of CO2 partial pressure and temperature on photosynthesis and calcification in a scleractinian coral. Glob Chang Biol. 2003;9: 1660–1668. 10.1046/j.1529-8817.2003.00678.x

[39] Hughes TP, Kerry JT, Baird AH, Connolly SR, Chase TJ, Dietzel A, et al Global warming impairs stock–recruitment dynamics of corals. Nature. 2019;568: 387–390. 10.1038/s41586-019-1081-y 30944475

[40] Doropoulos C, Ward S, Diaz-Pulido G, Hoegh-Guldberg O, Mumby PJ. Ocean acidification reduces coral recruitment by disrupting intimate larval-algal settlement interactions. Ecol Lett. 2012;15: 338–346. 10.1111/j.1461-0248.2012.01743.x 22321314

[41] Darling ES, Côté IM. Seeking resilience in marine ecosystems. Science (80-). 2018;359: 986–987. 10.1126/science.aas9852 29496864

[42] Darling ES, Mcclanahan TR, Côté IM. Combined effects of two stressors on Kenyan coral reefs are additive or antagonistic, not synergistic. Conserv Lett. 2010;3: 122–130. 10.1111/j.1755-263X.2009.00089.x

[43] Barshis DJ, Ladner JT, Oliver TA, Seneca FO, Traylor-Knowles N, Palumbi SR. Genomic basis for coral resilience to climate change. Proc Natl Acad Sci. 2013;110: 1387–92. 10.1073/pnas.1210224110 23297204 PMC3557039

[44] Middlebrook R, Anthony KRN, Hoegh-Guldberg O, Dove S. Thermal priming affects symbiont photosynthesis but does not alter bleaching susceptibility in Acropora millepora. J Exp Mar Bio Ecol. 2012;432–433. 10.1016/j.jembe.2012.07.005

[45] Matz M V., Treml EA, Aglyamova G V., Bay LK. Potential and limits for rapid genetic adaptation to warming in a Great Barrier Reef coral. PLoS Genet. 2018;14: 1–19. 10.1371/journal.pgen.1007220 29672529 PMC5908067

[46] NASEM. A decision framework for interventions to increase the persistence and resilience of coral reefs. Washington DC: National Academy of Sciences, Engineering and Medicine; 2019 10.17226/25424

[47] Quigley KM, Bay LK, Oppen MJH. The active spread of adaptive variation for reef resilience. Ecol Evol. 2019;9: 11122–11135. 10.1002/ece3.5616 31641460 PMC6802068

[48] Walsworth TE, Schindler DE, Colton MA, Webster MS, Palumbi SR, Mumby PJ, et al Management for network diversity speeds evolutionary adaptation to climate change. Nat Clim Chang. 2019;9: 632–636. 10.1038/s41558-019-0518-5

[49] Smale DA, Wernberg T, Oliver ECJ, Thomsen M, Harvey BP, Straub SC, et al Marine heatwaves threaten global biodiversity and the provision of ecosystem services. Nat Clim Chang. 2019;9: 306–312. 10.1038/s41558-019-0412-1

[50] Gattuso J-P, Magnan A, Bille R, Cheung WWL, Howes EL, Joos F, et al Contrasting futures for ocean and society from different anthropogenic CO2 emissions scenarios. Science. 2015;349: aac4722 10.1126/science.aac4722 26138982

[51] van Oppen MJH, Gates RD, Blackall LL, Cantin N, Chakravarti LJ, Chan WY, et al Shifting paradigms in restoration of the world’s coral reefs. Glob Chang Biol. 2017;23: 3437–3448. 10.1111/gcb.13647 28247459

[52] Oppen MJH Van Oliver JK, Putnam HM Gates RD. Building coral reef resilience through assisted evolution. Proc Natl Acad Sci. 2015;112: 2307–2313. 10.1073/pnas.1422301112 25646461 PMC4345611

[53] Anthony K, Bay LK, Costanza R, Firn J, Gunn J, Harrison P, et al New interventions are needed to save coral reefs. Nat Ecol Evol. 2017;1: 1420–1422. 10.1038/s41559-017-0313-5 29185526

[54] NASEM. A research review of interventions to increase the persistence and resilience of coral reefs. Natl Acad Sci Eng Med. Washington DC; 2019 10.17226/25279

[55] Hardisty PE, Roth CH, Silvery P, Mead D, Anthony K. Investment Case: A report provided to the Australian Government from the Reef Restoration and Adaptation Program. 2019.

[56] Hammond JS, Keeney RL, Raiffa H. Smart choices: a practical guide to making better life decisions. Boston: Harvard Business School Press; 1999.

[57] Gregory R, Failing L, Harstone M, Long G, McDaniels T, Ohlson D. Structured decision making: a practical guide to environmental management choices. West Sussex, UK: Wiley-Blackwell; 2012.

[58] Runge MC. An introduction to adaptive management for threatened and endangered species. J Fish Wildl Manag. 2011;2: 220–233. 10.3996/082011-JFWM-045

[59] Pandolfi JM, Bradbury RH, Sala E, Hughes TP, Bjorndal KA, Cooke RG, et al Global trajectories of the long-term decline of coral reef ecosystems. Science. 2003;301: 955–958. 10.1126/science.1085706 12920296

[60] Graham NAJ, Cinner JE, Norström A V., Nyström M. Coral reefs as novel ecosystems: embracing new futures. Curr Opin Environ Sustain. 2014;7: 9–14. 10.1016/j.cosust.2013.11.023

[61] Hicks CC, Cinner JE, Stoeckl N, McClanahan TR. Linking ecosystem services and human-values theory. Conserv Biol. 2015;29: 1471–1480. 10.1111/cobi.12550 26129942

[62] Stoeckl N, Hicks CC, Mills M, Fabricius K, Esparon M, Kroon F, et al The economic value of ecosystem services in the Great Barrier Reef: Our state of knowledge. Ann N Y Acad Sci. 2011;1219: 113–133. 10.1111/j.1749-6632.2010.05892.x 21332495

[63] Tallis H, Kareiva P, Marvier M, Chang A. An ecosystem services framework to support both practical conservation and economic development. Proc Natl Acad Sci U S A. 2008;105: 9457–9464. 10.1073/pnas.0705797105 18621702 PMC2474523

[64] Kareiva P, Watts S, McDonald R, Boucher T. Domesticated nature: shaping landscapes and ecosystems for human welfare. Science. 2007;316: 1866–1869. 10.1126/science.1140170 17600209

[65] Hobbs RJ, Higgs E, Hall CM, Bridgewater P, Chapin FS, Ellis EC, et al Managing the whole landscape: Historical, hybrid, and novel ecosystems. Front Ecol Environ. 2014;12: 557–564. 10.1890/130300

[66] Hobbs RJ, Higgs E, Harris JA. Novel ecosystems: implications for conservation and restoration. 2009; 599–605. 10.1016/j.tree.2009.05.012 19683830

[67] Kaebnick GE, Heitman E, Collins JP, Delborne JA, Landis WG, Taneyhill LA, et al Precaution and governance of emerging technologies. Science. 2017;354: 710–711. doi: 10.1126/science.aah5125 11 10.1126/science.aah5125 27846595

[68] Hussey K, Dovers S. Uncertainty: Risk, technology and the future. In: Harris PG, editor. Routledge handbook of global environmental politicspolitics. London, United Kingdom: Routledge; 2014 pp. 231–245.

[69] Webber BL, Raghu S, Edwards OR. Opinion: Is CRISPR-based gene drive a biocontrol silver bullet or global conservation threat? Proc Natl Acad Sci. 2015;112: 10565–10567. 10.1073/pnas.1514258112 26272924 PMC4553820

[70] Scott JK, McKirdy SJ, Merwe J Van Der, Green R, Burbidge AA, Pickles G, et al Zero-tolerance biosecurity protects high-conservation-value island nature reserve. Sci Rep. 2017;7: 1–9. 10.1038/s41598-016-0028-x 28396608 PMC5428405

[71] Mankins JC. Technology readiness assessments: a retrospective. Acta Astronaut. 2009;65: 1216–1223. 10.1016/j.actaastro.2009.03.058

[72] Dimasi JA, Feldman L, Seckler A, Wilson A. Trends in risks associated with new drug development: Success rates for investigational drugs. Clin Pharmacol Ther. 2010;87: 272–277. 10.1038/clpt.2009.295 20130567

[73] Kaitin KI, Dimasi JA. Pharmaceutical innovation in the 21st century: New drug approvals in the first decade, 2000–2009. Clin Pharmacol Ther. 2011;89: 183–188. 10.1038/clpt.2010.286 21191382

[74] Kaur K, Rampersad G. Trust in driverless cars: Investigating key factors influencing the adoption of driverless cars. J Eng Technol Manag—JET-M. 2018;48: 87–96. 10.1016/j.jengtecman.2018.04.006

[75] Martin TG, Nally S, Burbidge AA, Arnall S, Garnett ST, Hayward MW, et al Acting fast helps avoid extinction. Conserv Lett. 2012;5: 274–280. 10.1111/j.1755-263X.2012.00239.x

[76] Iacona GD, Possingham HP, Bode M. Waiting can be an optimal conservation strategy, even in a crisis discipline. Proc Natl Acad Sci. 2017;114: 201702111 10.1073/pnas.1702111114 28894004 PMC5625895

[77] Prober SM, Doerr VAJ, Broadhurst LM, Williams KJ, Dickson F. Shifting the conservation paradigm: a synthesis of options for renovating nature under climate change. Ecol Monogr. 2019;89: 1–23. 10.1002/ecm.1333

[78] Hoegh-Guldberg O, Hughes L, McIntyre S, Lindenmayer DB, Parmesan C, Possingham HP, et al Assisted colonization and rapid climate change. Science. 2008;321: 345–346. 10.1126/science.1157897 18635780

[79] Ricciardi A, Simberloff D. Assisted colonization is not a viable conservation strategy. Trends Ecol Evol. 2009;24: 248–253. 10.1016/j.tree.2008.12.006 19324453

[80] Straub J. In search of technology readiness level (TRL) 10. Aerosp Sci Technol. 2015;46: 312–320. 10.1016/j.ast.2015.07.007

[81] Charles C. Shared decision-making in the medical encounter: what does it mean? (or it takes at least two to tango). Soc Sci Med. 1997;44: 681–692. 10.1016/s0277-9536(96)00221-3 9032835

[82] Possingham HP, Bode M, Klein CJ. Optimal conservation outcomes require both restoration and protection. PLOS Biol. 2015;13: e1002052 10.1371/journal.pbio.1002052 25625277 PMC4308106

[83] Jones HP, Jones PC, Barbier EB, Blackburn RC, Rey Benayas JM, Holl KD, et al Restoration and repair of Earth’s damaged ecosystems. Proc R Soc B Biol Sci. 2018;285 10.1098/rspb.2017.2577 29491171 PMC5832705

[84] Dixon GB, Davies SW, Aglyamova GA, Meyer E, Bay LK, Matz M V. Genomic determinants of coral heat tolerance across latitudes. Science. 2015;348: 1460–1462. 10.1126/science.1261224 26113720

[85] Riginos C, Hock K, Matias AM, Mumby PJ, van Oppen MJH, Lukoschek V. Asymmetric dispersal is a critical element of concordance between biophysical dispersal models and spatial genetic structure in Great Barrier Reef corals. Divers Distrib. 2019;25: 1684–1696. 10.1111/ddi.12969

[86] Lough JM, Anderson KD, Hughes TP. Increasing thermal stress for tropical coral reefs: 1871–2017. 2018; 1–8. 10.1038/s41598-018-24530-9 29666437 PMC5904187

[87] Boström-Einarsson L, Babcock RC, Bayraktarov E, Ceccarelli D, Cook N, Ferse SCA, et al Coral restoration—A systematic review of current methods, successes, failures and future directions. PLoS One. 2020;15: e0226631 10.1371/journal.pone.0226631 31999709 PMC6992220

[88] Cameron KA, Harrison PL. Density of coral larvae can influence settlement, post-settlement colony abundance and coral cover in larval restoration. Sci Rep. 2020;10: 5488 10.1038/s41598-020-62366-4 32218470 PMC7099096

[89] Davies RJ, Kat HM, Lu S. Fund of hedge funds portfolio selection: A multiple-objective approach. J Deriv Hedge Funds. 2009;15: 91–115. 10.1057/jdhf.2009.1

[90] Kaiser C. NatureVest: natural capital investment solutions to transform the way we protect nature. Soc Res (New York). 2012;82: 749–760. doi: muse.jhu.edu/article/603160.

[91] Joseph LN, Maloney RF, Possingham HP. Optimal allocation of resources among threatened species: a project prioritization protocol. Conserv Biol. 2009;23: 328–338. 10.1111/j.1523-1739.2008.01124.x 19183202

[92] Bottrill MC, Joseph LN, Carwardine J, Bode M, Cook C, Game ET, et al Is conservation triage just smart decision making? Trends Ecol Evol. 2008;23: 649–654. 10.1016/j.tree.2008.07.007 18848367

[93] Wilson KA, Law EA. Ethics of conservation triage. Front Ecol Evol. 2016;4: 1–8. 10.3389/fevo.2016.00112

[94] Commonwealth_of_Australia. Reef 2050 Long-Term Sustainability Plan. Canberra, Australia; 2018.

[95] Kennedy E V., Perry CT, Halloran PR, Iglesias-Prieto R, Schoenberg CHL, Wisshak M, et al Avoiding coral reef functional collapse requires local and global action. Curr Biol. 2013;23: 912–918. 10.1016/j.cub.2013.04.020 23664976

[96] Gascoigne J, Lipcius RN. Allee effects in marine systems. Mar Ecol Ser. 2004;269: 49–59. 10.3354/meps269049

[97] Taylor CM, Hastings A. Allee effects in biological invasions. Ecol Lett. 2005;8: 895–908. 10.1111/j.1461-0248.2005.00787.x

[98] Curnock M, Marshall N, Tobin R, Stone-Jovicich S, Bohensky E, Pert P, et al. The Social and Economic Long Term Monitoring Program (SELTMP) 2014, Tourismin the Great Barrier Reef. Report to the National Environmental Research Program. Cairns; 2014.

[99] Hock K, Wolff NH, Ortiz JC, Condie SA, Anthony KRN, Blackwell PG, et al Connectivity and systemic resilience of the Great Barrier Reef. PLoS Biol. 2017;15: e2003355 10.1371/journal.pbio.2003355 29182630 PMC5705071

[100] Mumby PJ, Elliott IA, Eakin CM, Skirving W, Paris CB, Edwards HJ, et al Reserve design for uncertain responses of coral reefs to climate change. Ecol Lett. 2011;14: 132–140. 10.1111/j.1461-0248.2010.01562.x 21105980

[101] Bode M, Bode L, Armsworth PR. Larval dispersal reveals regional sources and sinks in the Great Barrier Reef. Mar Ecol Prog Ser. 2006;308: 17–25.

[102] Beyer HL, Cinner JE, Kennedy E V, Darling ES, Wilson KA, Beger M, et al Risk-sensitive planning for conserving coral reefs under rapid climate change. 2018; 1–10. 10.1111/conl.12587

[103] Torda G, Donelson JM, Aranda M, Barshis DJ, Bay L, Berumen ML, et al Rapid adaptive responses to climate change in corals. Nat Clim Chang. 2017;7: 627–636. 10.1038/nclimate3374

[104] Wise RM, Fazey I, Stafford Smith M, Park SE, Eakin HC, Archer Van Garderen ERM, et al Reconceptualising adaptation to climate change as part of pathways of change and response. Glob Environ Chang. 2014;28: 325–336. 10.1016/j.gloenvcha.2013.12.002

[105] Mantyka-Pringle CS, Martin TG, Rhodes JR. Interactions between climate and habitat loss effects on biodiversity: A systematic review and meta-analysis. Glob Chang Biol. 2012;18: 1239–1252. 10.1111/j.1365-2486.2011.02593.x

[106] Groves CR, Game ET. Conservation planning—informed decisions for a healthier planet. Greenwoord Village, Colorado: Roberts and Company Publishers; 2016.

[107] Hammond JS, Keeney RL, Raiffa H, Hammond BYJS, Keeney RL, Raiffa H. The Hidden Traps in Decision Making. Harv Bus Rev. 2006;January: 1–10. 10185432

[108] Beck U. Risk society: Towards a new modernity. London: Sage; 1992.

[109] Walton CJ, Hayes NK, Gilliam DS. Impacts of a regional, multi-year, multi-species coral disease outbreak in Southeast Florida. Front Mar Sci. 2018;5: 1–14. 10.3389/fmars.2018.00043 29552559 PMC5851660

[110] Precht WF, Gintert BE, Robbart ML, Fura R, Van Woesik R. Unprecedented Disease-Related Coral Mortality in Southeastern Florida. Sci Rep. 2016;6: 1–11. 10.1038/s41598-016-0001-8 27506875 PMC4979204

[111] Randall CJ, Van Woesik R. Some coral diseases track climate oscillations in the Caribbean. Sci Rep. 2017;7: 1–8. 10.1038/s41598-016-0028-x 28720811 PMC5515922

[112] Morrison TH, Adger N, Barnett J, Brown K, Possingham H, Hughes T. Advancing Coral Reef Governance into the Anthropocene. One Earth. 2020;2: 64–74. 10.1016/j.oneear.2019.12.014

[113] Fidelman P, McGrath C, Newlands M, Dobbs K, Jago B, Hussey K. Regulatory implications of coral reef restoration and adaptation under a changing climate. Environ Sci Policy. 2019; 1–9. 10.1016/j.envsci.2019.04.016

[114] Linkov I, Trump BD, Anklam E, Berube D, Boisseasu P, Cummings C, et al Comparative, collaborative, and integrative risk governance for emerging technologies. Environ Syst Decis. 2018;38: 170–176. 10.1007/s10669-018-9686-5 PMC10569133 37829286

